# Influence of family parenting style on the formation of eating behaviors and habits in preschool children: The mediating role of quality of life and nutritional knowledge

**DOI:** 10.1371/journal.pone.0288878

**Published:** 2023-07-20

**Authors:** Wang Ningning, Cheng Wenguang

**Affiliations:** 1 School of Physical Education, Liaoning Normal University, Dalian, China; 2 Graduate Students’ Affairs Department, Shenyang Sport University, Shenyang, China; 3 School of Management and Journalism, Shenyang Sport University, Shenyang, China; Zagazig University Faculty of Human Medicine, EGYPT

## Abstract

To provide empirical support for understanding the effects of different family parenting styles on the development of preschool children’s eating habits and to promote healthy child development. Using a randomized whole-group sampling method, full-time public kindergartens in three regions of China were selected as the study population of preschool children, and 1141 children’s guardians in these regions were surveyed and evaluated. It was used to examine the differential effects of different family parenting styles (EMBU) on preschool children’s eating behavior (CEBQ), while quality of survival (QLSCA) and nutritional literacy (NLS) played a mediating role in the process. The results showed that at the direct effect level, authoritative, authoritarian, coddling, and neglectful family parenting styles had significant effects on preschool children’s eating behavior (-0.161 ≤ β ≤ 0.232, p < 0.05); at the indirect effect level, family survival quality (QLSCA), and nutritional literacy (NLS) under the influence of different family parenting styles (EMBU) on children’s eating the total indirect effect was [OR] 0.273, 95%: CI 0.181–0.368. It is evident that it is imperative to develop good eating behaviors in children at preschool age.

## 1. Introduction

The age of 3–6 years is a critical period not only for the physical and mental development of preschool children but also for the formation of their eating behaviors [1]. Children’s eating behaviors are influenced by a combination of factors, including family environment, feeding style, and social environment. As an important arena for child growth and development, the family parenting environment plays an important role in the development of eating behaviors in preschool children [2]. Family parenting style incorporates the attitudes and behaviors of parents and other guardians, and it is considered a global measure of parental enthusiasm and control over children’s behavior [3]. Research shows that the type of family parenting style affects the way children respond to problems and that parental feeding style is a major determinant of children’s eating behavior, primarily through changes in food availability and social environment [4]. For example, the overall diet quality of preschool children in the United States is low [5], primarily due to higher than recommended intakes of refined grains, solid fats, and added sugars. If improper feeding and poor eating behaviors occur over time, the healthy development of children can be affected [6]. Chinese parents and grandparents are the primary caregivers of preschool children, and different parenting styles have differential effects on preschool children’s eating behaviors. For example, grandparents often have conflicting ideas about child feeding with their parents, and grandparents may ignore the dietary rules set by their parents for their children or conceal the fact that their parents are providing high-calorie, high-fat foods to their children [7].

Parents are the initial food providers and controllers of children [8]. Family parenting practices are significantly related to children’s eating behaviors, dietary preferences, food choices, and childhood overweight or obesity, implying that poor parental feeding behaviors play a role in the development of eating behavior problems in preschool-aged children [9, 10]. Baumrind found in a study of 100 European-American middle-class families that parents have different levels of influence on their children’s lives in four areas: expressive attitudes, disciplinary methods, communication, and expectations. Based on the stringency of the requirements, family parenting styles can be classified into three types: authoritative, authoritarian, and coddling [11]. Maccoby and Martin added the dimension of "support" to this. There four types of parenting styles are [12, 13]. The study showed that different family parenting styles (EMBU) have different effects on adolescent children’s eating behaviors (CEBQ). For example, authoritarian parenting styles usually have strict requirements on meal speed, eating preferences, and picky eating behaviors, which can increase children’s eating independence and normality but also limit children’s initiative in food or object selection to some extent. Parents with a democratic parenting style encourage children to think independently and make their own choices, which helps children develop positive eating behaviors such as independent eating and balanced meals while cultivating eating habits. The coddling parenting style gives children the right to make food choices, but the lack of reasonable guidance and regulation can easily lead to overeating, picky eating, and partial eating behaviors [14].

In conclusion, preschool children are in a vital period for forming excellent eating habits, and family parenting style is an important arena for this development. Existing research has concentrated on the influence of family parenting styles and parental feeding behaviors on children’s eating behaviors, ignoring the fact that different family parenting styles determine the diversity of parents’ attitudes toward life, which affects the overall quality and level of survival of the family as a whole, and thus affects preschool children’s eating behaviors. This study aims to investigate the mechanism of family parenting style on preschool children’s eating behavior by constructing a mediation model, and to explain the chain mediation effect of family survival quality and nutritional literacy in this process, so as to provide theoretical reference for the development of good eating behavior in children.

## 2. Objects and methods

### 2.1. Object

A random cluster sampling method was used to select three regions in China: Shanghai, Beijing, and Guangzhou, and two full-day kindergartens were selected in each region. Specifically, from September to October 2022, the guardians of children in these areas were surveyed. Inclusion criteria: (1) preschool children aged 3–6 years; (2) primary caregivers willing to cooperate with this survey. Exclusion criteria: (1) children with chronic gastritis, constipation, or other chronic diseases that affect children’s appetite or food intake, or may cause changes in children’s eating behavior in the past two months; (2) children’s caregivers with communication problems. Primary caregivers were defined as family members who were responsible for the child’s three meals a day and daily living, including parents, grandparents, relatives, or babysitters. A total of 1141 valid questionnaires were obtained, with an effective rate of 92.5%, excluding those with an omission rate of >30% and incomplete questionnaires. Among them, 551 (48.291%) were boys and 590 (52.709%) were girls; the mean age was (4.49±0.99) years. The study was approved by the Ethics Committee of Liaoning Normal University (approval number: 20220629002), and all participants signed an informed consent form.

### 2.2. Methodology

#### 2.2.1. Family Parenting Style Inventory (EMBU)

The Family Parenting Style Scale (EMBU) was developed by Swedish clinical psychologist Perris and revised by Chinese scholars Yang LZ and Yue DM to form the EMBU in this paper, which includes four types of family parenting styles: authoritative, authoritarian, doting, and neglectful [15]. The entries were rated on a 7-point Likert scale, with higher scores indicating more frequent use of the corresponding parenting behaviors by parents. The questionnaire’s Cronbach’s a coefficient was 0.872, its KMO was 0.851, and its Bartlett’s sphericity test was 10440.714, p = 0.000, indicating that it has good reliability and validity and can measure the actual situation [16].

#### 2.2.2. Quality of Survival Scale (QLSCA)

Varni et al. (2001) developed the Child Survival Quality Measurement Scale (Peds QL 4.0) [17], which was localized by Chinese scholars Lu YY et al. (2008) [18]. Since the Child Survival Quality Scale contains several versions of the Self-Assessment Scale for Children of Different Ages (5 to <8, 8 to <13, and 13 to 18 years old) and the Parental Surrogate Scale for Children of Different Ages (2 to <5, 5 to <8, 8 to <13, and 13 to 18 years old), the dimensions and content of the entries are basically the same. In this study, children of different age groups were evaluated using a self-assessment scale that contains 4 dimensions of physical functioning (e.g., the family can provide timely medical care for the child’s body), emotional functioning (the child can get emotional support and encouragement at school or other places), social functioning (the child can get the social help and support he or she needs), and role functioning (helping the child learn to adapt to multiple roles), with each entry using the Likert 7-point scale, with higher scores indicating a better quality of survival. Since the factor loadings of the role function dimension were lower than the standard of 0.6, the QLSCA included 3 indicators after the deletion of the remeasurement dimension, and the scale had a Cronbach’s alpha coefficient of 0.862, a KMO of 0.737, and a Bartlett’s sphericity test of 1591.530, p = 0.000, indicating good reliability and validity [19].

#### 2.2.3. Nutrient Literacy Scale (NLS)

Nutritional literacy was used to assess respondents’ ability to understand nutritional information, and the questionnaire included three sections: nutritional knowledge, behavior, and skills. Among them, the nutrition knowledge section mainly covers conceptual knowledge about food and nutrition, such as "the effect of fried food on the body; the behavior and skills section mainly covers the ability to obtain, select, prepare, and consume food, as well as the preference of food, such as whether children "usually drink milk", "usually eat breakfast", "eat snacks", etc. "Do they usually eat breakfast", "Do they eat snacks", etc. The questions on nutrition knowledge and eating behaviors and skills are set differently in different nutrition literacy questionnaires. Combined with the recommendations of the Dietary Guidelines for Chinese Residents (2016), the entries were rated on a 7-point Likert scale, with higher scores indicating better nutritional literacy in children. The Cronbach’s alpha coefficient for each dimension of the scale was 0.895, the KMO was 0.750, and the Bartlett’s sphericity test value was 2033.419, p = 0.000, indicating good reliability and validity of the questionnaire and a stable structure of factor analysis.

#### 2.2.4. Eating Behavior Scale (CEBQ)

The Eating Behavior Measure is a questionnaire used to evaluate the characteristics of children’s eating behaviors and appetites, mainly through their parents’ perceptions of various behaviors during eating [20]. The Eating Behavior Measurement Scale for Children includes three dimensions: emotional eating, exogenous eating, and avoidance eating (also known as picky eating in children) [21]. Emotional eating behavior refers to children’s eating behavior to reduce negative emotions such as anxiety, depression, and anger in the absence of hunger [22]; exogenous eating behavior refers to children’s eating behavior due to external stimuli such as the appearance and smell of food, regardless of the presence of gastric sensation; avoidance eating behavior refers to children’s intentional control of their daily dietary intake. The Likert 7-point scale was used for each item of the Child Eating Behavior Scale, ranging from "strongly disagree" to "strongly agree" on a scale of 1 to 7, with higher scores indicating more frequent eating behavior in adolescents. The Cronbach’s alpha coefficient for each dimension of the scale was 0.929, the KMO was 0.761, and the Bartlett’s sphericity test was 2769.758, p = 0.000, indicating good reliability and validity of the questionnaire and a stable structure of the factor analysis.

### 2.3. Quality control

Questionnaires were administered to preschool children and their parents in a face-to-face format, and the quality of the questionnaires was strictly controlled. Attention was paid to quality control in questionnaire design, investigator training, and field implementation, and study subjects were screened in strict accordance with inclusion and exclusion criteria. Data entry was double-entered and double-checked with logical verification, screening and elimination of outliers, and consistency requirements of 100%.

### 2.4. Statistical methods

Descriptive statistical analysis, correlation analysis, factor analysis, and mediating effect testing were performed with the help of SPSS 26.0 and AMOS 24.0. Specifically, descriptive statistical analysis, correlation analysis, and factor analysis were conducted with the help of SPSS 26.0 statistical software for family parenting style (EMBU) and eating behavior of preschool children (CEBQ), and Boostrop in AMOS24 was used to test for mediating effects for family survival quality (QLSCA) and nutritional literacy (NLS).

## 3. Research results

### 3.1. Test for common method deviation

Procedural and statistical controls were used to reduce and test the common method bias in this study. Procedural controls included (1) requiring respondents to fill out the questionnaire anonymously; (2) setting questionnaire screening criteria to eliminate regular and repetitive responses; (3) setting some reverse question items in the questionnaire design; and 4 setting different rating scales using each of the Richter scales. Statistically, Harman’s one-way test was used to conduct principal component factor analysis on all items, and the results showed that there were seven factors with eigenvalues greater than one, and the explanation rate of the first factor was 17.596%, which was much less than the critical standard of 40%, indicating that there was no serious common method bias in this study.

### 3.2. Descriptive statistics and correlation analysis

To investigate the correlation between different family parenting styles (EMBU) and children’s eating behaviors (CEBQ), this study used Kendall correlation analysis to statistically analyze the degree of correlation as well as the significance between each variable. The results of the study showed that the four family parenting styles, authoritative, authoritarian, doting, and neglectful, had good internal correlations (0.082 ≤ r ≤ 0.352, p ≤ 0.01). There was also a moderate correlation between family parenting style (EMBU) and children’s survival quality (QLSCA), nutritional literacy (CEBQ), and eating behavior (CEBQ) (0.115≤r≤0.383, p≤0.01), where authoritarian family parenting style was weakly correlated with children’s eating behavior (r = 0.023, p>0.05), but its low significance resulted in a non-significant correlation. Overall, there was a significant correlation between different family parenting styles (EMBU) and the eating behavior of preschool children (CEBQ), and there was good internal consistency as well as external correlation between the variables (Table 1).

**Table 1:** Kendall correlation coefficients between latent variables.

Projects	M±SD	Permissive	Authoritative	Neglectful	Authoritarian	NLS	QLSCA
Permissive	4.549±1.395	1.000					
Authoritative	5.042±1.127	0.190**	1.000				
Neglectful	5.068±1.338	0.082**	0.284**	1.000			
Authoritarian	4.881±1.205	0.102**	0.298**	0.352**	1.000		
NLS	4.892±0.973	0.242**	0.301**	0.294**	0.269**	1.000	
QLSCA	5.300±1.055	0.115**	0.332**	0.346**	0.383**	0.265**	1.000
CEBQ	4.412±1.521	0.023	0.241**	0.311**	0.269**	0.246**	0.269**

** Significant correlation at the 0.01 level (two-tailed).

### 3.3. Comparison of eating behavior at different ages

　 Analysis of variance (ANOVA) was used to examine the variability of eating behavior styles among children at different age stages. The chi-square test showed that family upbringing (EMBU), quality of survival (QLSCA), nutritional literacy (NLS), and eating behavior (CEBQ) were consistent across age stages (sig two-tailed), and each dimension could be tested for variance in the next step. The results of the ANOVA test showed that there was no significant difference (p > 0.05) between family parenting at different ages, which means that it is difficult to produce changes in the effects of family parenting style (EMBU) on children as they grow older. There were no significant differences (p > 0.05) in the quality of survival (QLSCA) and nutritional literacy (NLS) of children at different ages, which means that age changes have little impact on the lifestyle and nutritional structure of children (Table 2). The reason for this is that the quality of survival (QLSCA) and nutritional literacy (NLS) of preschool children are mainly supported by their parents and families, and children’s ability to make their own choices is limited. Finally, there were significant differences (p < 0.05) in eating behaviors among children at different ages, which means that children’s autonomous selectivity in emotional eating, exogenous eating, and avoidance eating increases with age. Overall, there were no significant differences between children at different ages in terms of family parenting (EMBU), quality of survival (QLSCA), and nutritional literacy (NLS), but there were significant differences at the level of eating behaviors.

**Table 2:** ANOVA analysis of eating behavior at different ages.

ANOVA	Mean Square	F	Significance
Project	1.629	0.837	0.474
Permissive	1.205	0.948	0.417
Authoritative	0.480	0.267	0.849
Neglectful	1.945	1.341	0.260
Authoritarian	0.806	0.851	0.466
NLS	0.355	0.318	0.812
QLSCA	1.127	2.661	0.047

### 3.4. Structural equation model testing

　　The soundness of the structural equation model is usually tested by its goodness-of-fit [23]. The goodness-of-fit output from AMOS 26.0 statistical software includes three types of absolute fit indices: value-added fit indices and absolute fit indices. The overall fit indices of the model in this study are shown in the following Table 3: the model fit reached a significant level at a 95% confidence interval of X^2^/df = 2.835 (P < 0.05), and the model fit values all met the requirements of the reference values, indicating that there is a good fit between the hypothetical model and the actual data, and that the model fits better overall.

**Table 3:** Study model fit.

Projects	Fitting index	Model Fitted Values		Explanation and Explanation
Absolute fit index	P-value	0.001	<0.05, indicating a good model fit	
	Square root of the squared average residual RMR	0.042	<0.05, indicating a good model fit	
	Goodness-of-fit index GFI	0.959	>0.90, indicating a good model fit	
	Adjusted goodness-of-fit index AGFI	0.945	>0.90,indicating a good model fit	
	Root Mean Square Error of Approximation RMSEA	0.040	<0.05, indicating a good model fit	
Value-added fitting index	Relative fit index CFI	0.820	>0.90, indicating a good model fit	
	Standard fit index NFI	0.973	>0.90, indicating a good model fit	
	Tucker-Lewis Index TLI	0.978	>0.90, indicating a good model fit	
	Incremental fitting index	0.982	>0.90, indicating a good model fit	
Parsimonious fit index	Simple effect specification fit index PNFI	0.792	>0.50, indicating a good model fit	
	Summary-effect comparative fit index PCFI	0.799	>0.50, indicating a good model fit	
	X^2^/df	2.835	1<NC<3, indicating good adaptation	

### 3.5. Hypothesis testing

**Fig 1:**
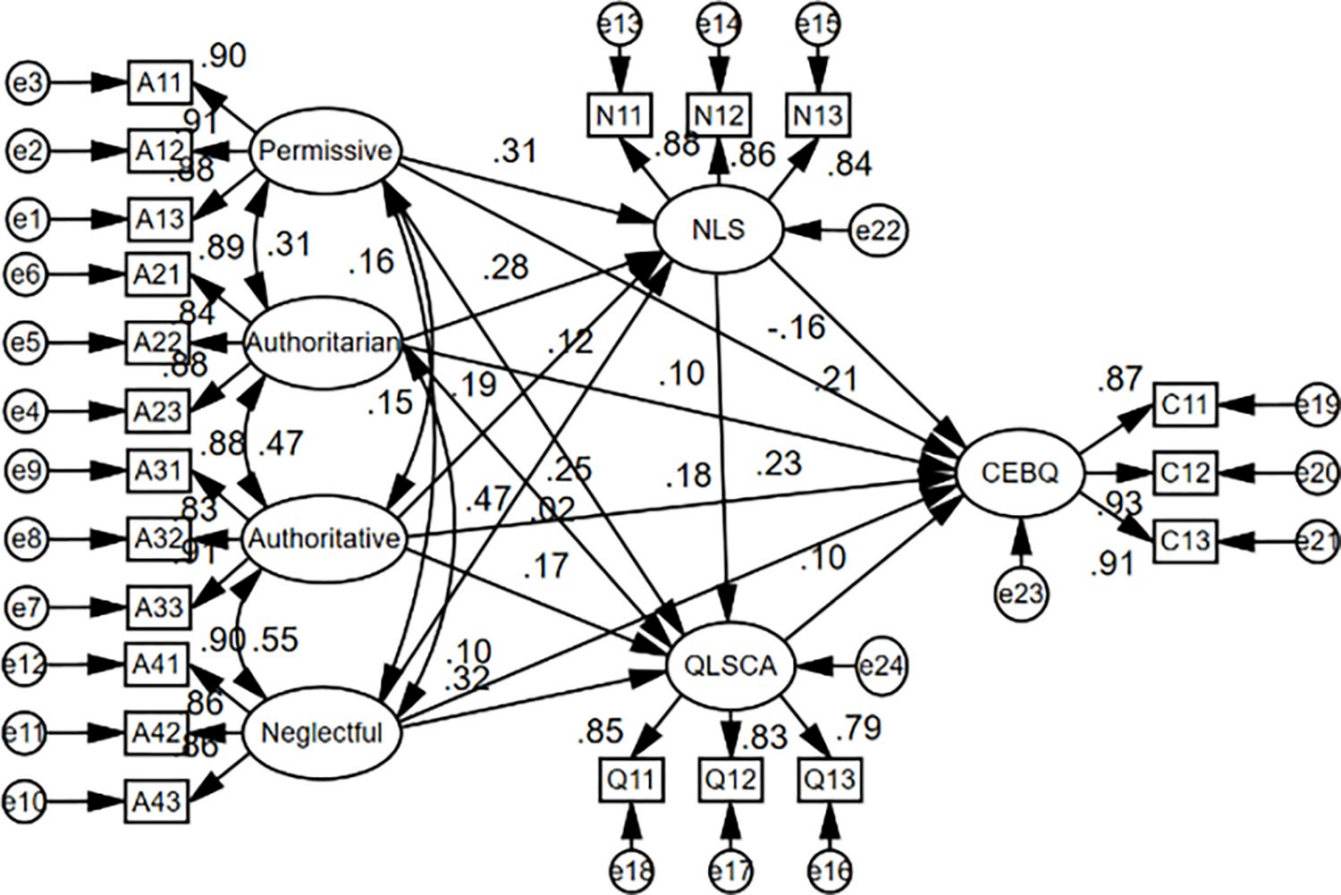
Structural equation model.

After goodness-of-fit tests, the structural equation model measurements are shown in Fig 1, and the solid lines represent paths that are significant at the P<0.05 level and above. In order to further examine the direct, indirect, and total effects among each latent variable of the most reasonable model, a comparison of the direct, indirect, and total effects of the most reasonable model path coefficients was conducted.

#### 3.5.1. Direct effect analysis

The direct effects of this study were mainly used to test the pathways of the effects of different family parenting styles (EMBU) on children’s eating behavior (CEBQ). In addition, the direct effects of child survival quality (QLSCA) and nutritional literacy (NLS) need to be tested before testing their mediating roles. Table 4 shows the standardized path coefficients between latent variables and their corresponding P-value values. The results of the study showed that different family parenting styles (EMBU) had differential effects on children’s eating behavior (CEBQ) (-0.161 ≤ β ≤ 0.232), and the ranking of their effects on preschool children’s eating behavior was authoritative (β = 0.232, p < 0.01), authoritarian (β = 0.097, p = 0.013), neglectful (β = 0.096, p = 0.016), and coddling (β = -0.161, p<0.01), but authoritarian and neglectful family parenting styles had a non-significant effect on children’s eating behaviors.

**Table 4:** Path test results.

Path way	Estimate	S.E.	C.R.	S-Estimate	P
Permissive→NLS	0.229	0.022	10.544	0.299	***
Authoritative→NLS	0.145	0.027	5.463	0.188	***
Neglectful→NLS	0.110	0.032	3.489	0.120	***
Authoritarian→NLS	0.254	0.032	7.946	0.270	***
Permissive→QLSCA	0.018	0.019	0.918	0.026	0.359
Authoritative→QLSCA	0.118	0.023	5.093	0.171	***
Neglectful→QLSCA	0.261	0.028	9.337	0.318	***
NLS→QLSCA	0.158	0.03	5.248	0.176	***
Authoritarian→QLSCA	0.215	0.029	7.491	0.254	***
NLS→CEBQ	0.289	0.053	5.467	0.208	***
QLSCA→CEBQ	0.152	0.071	2.15	0.098	0.032
Permissive→CEBQ	-0.172	0.034	-5.092	-0.161	***
Authoritative→CEBQ	0.249	0.041	6.129	0.232	***
Neglectful→CEBQ	0.123	0.051	2.418	0.096	0.016
Authoritarian→CEBQ	0.126	0.051	2.480	0.097	0.013

### 3.5.2. Indirect effect analysis

In this paper, the indirect effects self-help method was used to test for mediating effects, and the indirect effects self-help method can produce confidence intervals for indirect effects with statistical test power, especially bias-corrected bootstrapping (BCB), and the test results are shown in Table 5. Different family parenting styles (EMBU) produced mediating effects on children’s eating behavior (CEBQ) through family survival quality (QLSCA), with a total indirect effect of [OR] 0.273, 95% CI0.181–0.368, according to the results of the Bootstrap mediated effect test. The mediated effect of survival quality (QLSCA) on children’s eating behavior was 0.182 (p<0.05), the mediated effect value of nutritional literacy (NLS) on children’s eating behavior was 0.085 (p<0.05), and the chain mediated effect value of survival quality (QLSCA) and nutritional literacy (NLS) on children’s eating behavior was 0.005 (p<0.05). It is evident that survival quality (QLSCA) and nutritional literacy (NLS) have significant mediating effects on the eating behavior of preschool children (CEBQ) under the influence of different family parenting styles (EMBU).

**Table 5:** Bootstrap-mediated effects test.

Paths	Effect Value	Boot SE	P	Bias-Corrected 95%CI		Percentile 95%CI	
				lower	Upper	lower	Upper
Overall effect	0.599	0.051	0.001	0.498	0.698	0.504	0.700
Direct effect	0.326	0.082	0.001	0.165	0.326	0.166	0.490
Direct effect	0.273	0.048	0.001	0.179	0.273	0.181	0.368
int_1	0.062	0.013	0.001	0.038	0.090	0.038	0.090
int_2	0.056	0.015	0.001	0.031	0.088	0.032	0.091
int_3	0.039	0.01	0.001	0.020	0.059	0.021	0.061
int_4	0.025	0.009	0.001	0.010	0.044	0.011	0.046
int_5	0.029	0.014	0.027	0.003	0.059	0.005	0.060
int_6	0.026	0.013	0.023	0.002	0.054	0.005	0.056
int_7	0.018	0.009	0.020	0.002	0.038	0.004	0.040
int_8	0.012	0.007	0.017	0.001	0.028	0.002	0.031
int_9	0.005	0.002	0.019	0.000	0.010	0.001	0.011

Note: int_1–4 are mediating effects of survival quality; int_5–8 are mediating effects of nutrient literacy; int_9 are chain mediating effects of survival quality, nutrient literacy.

## 4. Research discussion

### 4.1. Analysis of eating behaviors and characteristics of preschool children

Picky eating, anorexia, refusal to eat, or other eating behaviors that occur in children aged 3–7 years without organic diseases when food supply is adequate and the caregiver has normal feeding ability are examples of preschool children’s eating behavior [24]. The preschool period is a critical period for children’s physical and mental development, as well as an initiation period for the development of eating habits. With the continuous improvement of living standards in countries around the world, the dietary structure and eating behaviors of children at different stages of development are also changing significantly, and the eating behaviors and characteristics of preschool children will have many effects on their later growth. At present, the issue of preschool children’s eating behavior has become a hot topic of global concern. According to relevant research data, eating behavior problems are a common developmental behavior problem in preschool children, and the prevalence of eating behavior problems in children is high worldwide. For example, the prevalence of eating behavior problems among normal children in western developed countries ranges from 30% to 45% [25], while the prevalence of eating behavior problems among children in some Asian countries ranges from 39.7% to 65.1% [26]. It can be seen that preschool children are an important period for the formation and development of their eating behaviors, and it is also the best time for eating behavior interventions [27].

In this study, a comparison of eating behaviors of children at different ages revealed a significant difference in eating behaviors (CEBQ) between the different stages (F = 2.661, p<0.05). This finding is in line with previous studies, which concluded that the development of eating behavior in preschool children is not completely linear, and there is a regression in eating behavior at a certain age, but overall there is more room for preschool children to add value. The reason for this is that preschoolers are younger and their ability to eat actively is limited, so the formation of eating behaviors is more likely to be influenced by family and environmental factors. Studies have shown that family parenting styles, parental feeding behaviors, and meal combinations can influence the development of preschool children’s eating behaviors [28, 29]. EK et al. suggest that the age of 2–5 years is a period of high prevalence of eating behavior problems in children, where picky eating, irregular eating, and snacking are common [30, 31]. Healthy eating behaviors can provide a solid guarantee for the good nutritional status and physical and mental development of children and adolescents; while poor eating behaviors in children not only lead to many problems such as growth retardation, malnutrition, neuromotor disorders and psycho-emotional abnormalities, but also have a close relationship with the occurrence of chronic non-communicable diseases such as hypertension, coronary heart disease and diabetes in adulthood [32]. Therefore, it has become important to study the status of eating behavior habits of preschool children and the factors influencing them, and to cultivate good eating behavior in children.

### 4.2. Analysis of the direct effect of family parenting style on preschool children’s eating behavior

Family parenting style is a relatively stable behavioral style and tendency that parents exhibit in the course of educating and raising their children on a daily basis. Different family parenting styles have differential effects on preschoolers’ eating behaviors [33]. Musher (2013) showed that family feeding behavior is significantly associated with children’s eating behavior, and poor feeding behavior leads to eating behavior problems in children [9]. For example, authoritarian parenting styles are usually more demanding on preschoolers, often with mandatory behavioral requirements for meal speed, table behavior, and picky eating; authoritative parenting styles not only improve children’s meal independence, but also develop good eating habits and meal speed, but to some extent limit preschoolers’ free choice of food; coddling is to allow children to make their own decisions about food. The coddling type is to give preschoolers the full right to choose food according to their own developmental level, but this type of parenting is likely to lead to excessive eating and picky eating behavior; the neglect type of parenting is less effective in promoting children’s eating behavior, and is mainly found in families with a large number of children or in remote rural areas. As the earliest place for young children to develop eating behaviors, the influence of family on preschool children’s eating behaviors should not be underestimated.

**Fig 2:**
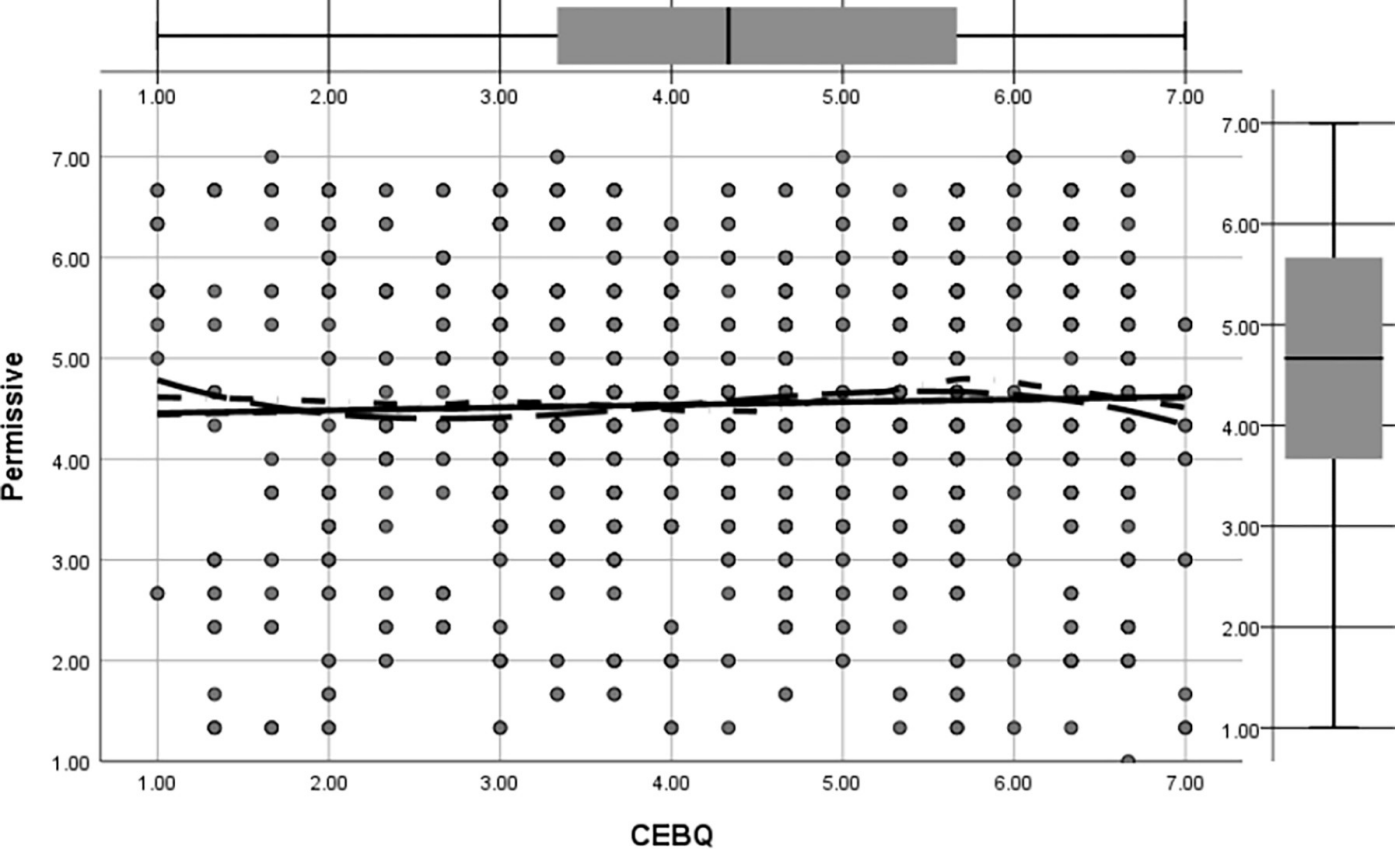
Regression images of the effect of permissive parenting style on preschool children’s eating behavior.

**Fig 3:**
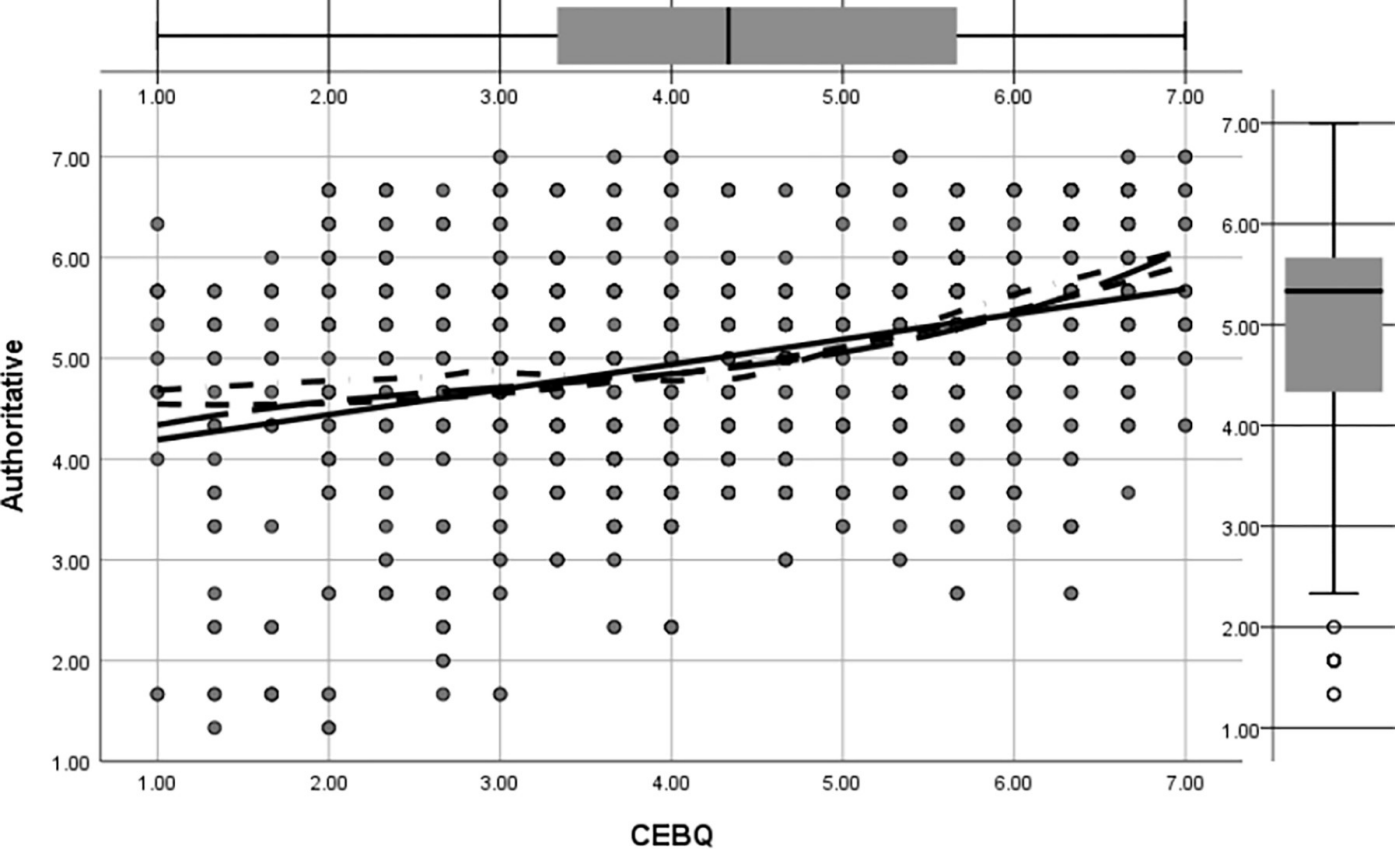
Regression images of the effect of authoritative parenting style on preschool children’s eating behavior.

**Fig 4:**
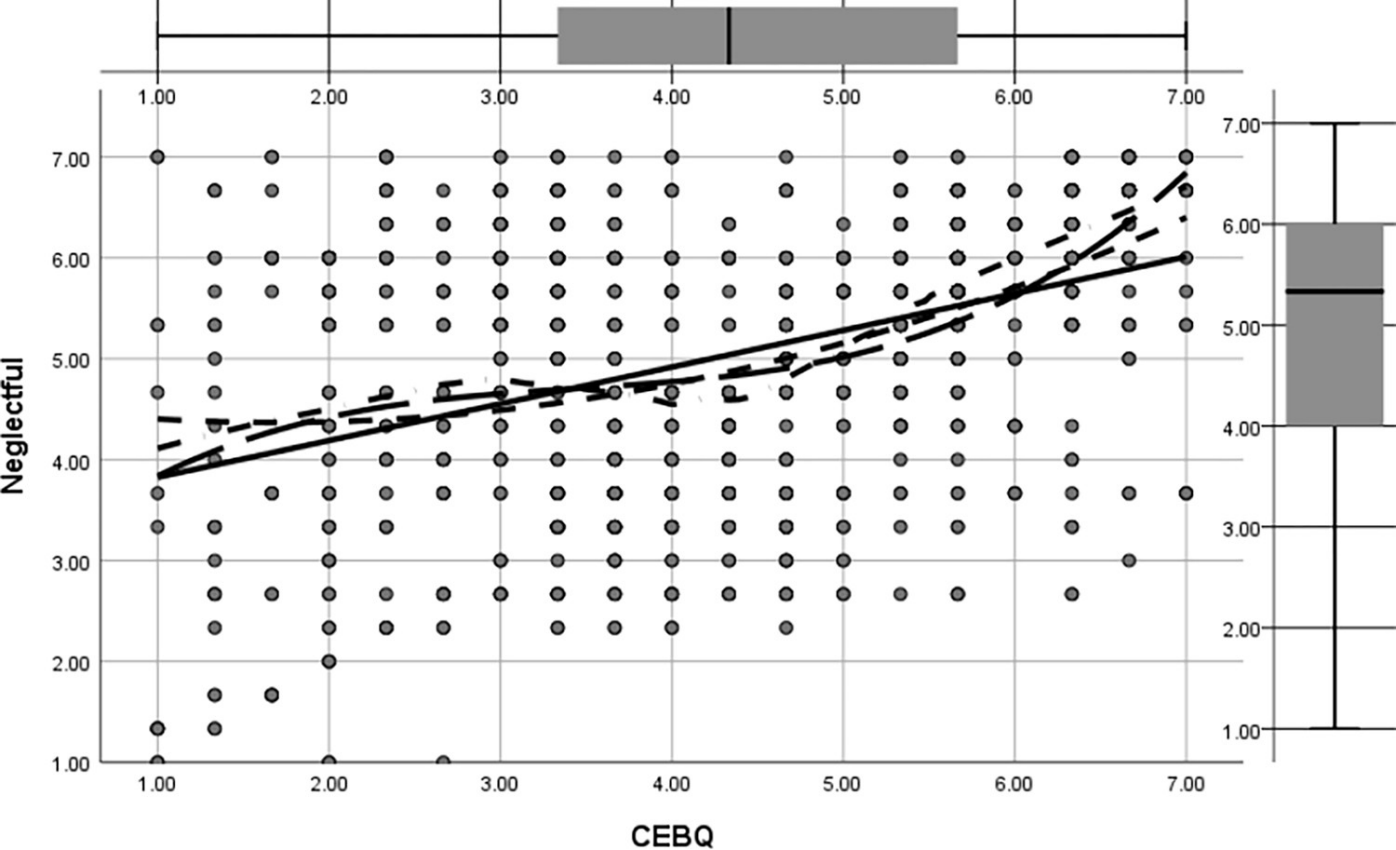
Regression image of the effect of neglectful parenting style on preschool children’s eating behavior.

**Fig 5:**
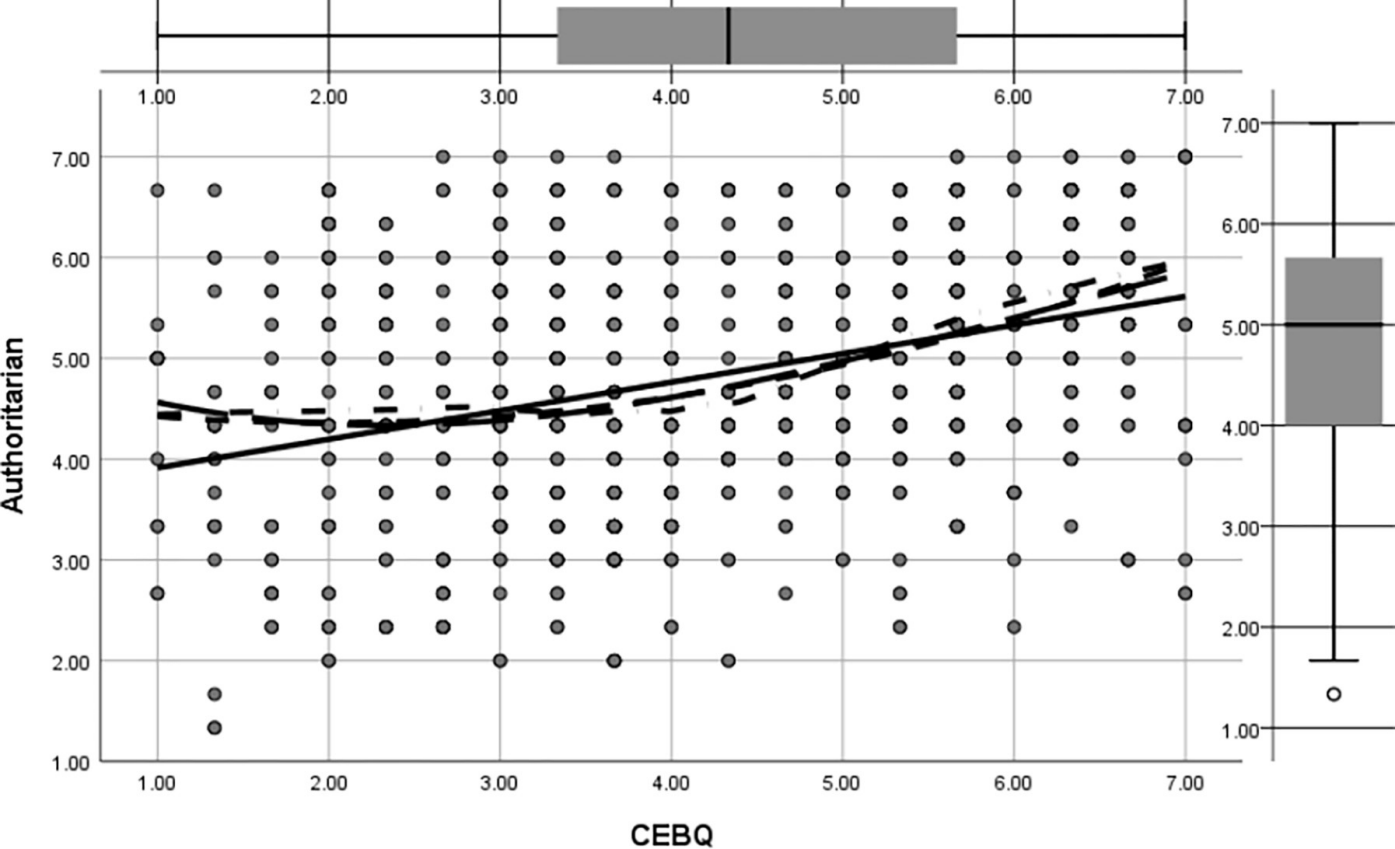
Regression of the effect of authoritarian parenting style on preschool children’s eating behavior.

Figs 2 to 5 shows the regression pathways of authoritative, authoritarian, patronizing, and neglectful family parenting style (EMBU) on children’s eating behavior (CEBQ) from left to right. In this study, the four main types of family parenting styles were authoritative, authoritarian, coddling, and neglectful, and their direct effects on preschool children’s eating behaviors (β = 0.326, p<0.05) were more significant. Fig 2 shows the direct effect of permissive parenting style on preschool eating behavior was (β = -0.161, p<0.05); Fig 3 shows the direct effect of authoritative parenting style on preschoolers’ eating behavior was (β = 0.232, p<0.05); Fig 4 shows the direct effect of neglectful parenting style on preschool children’s eating behavior was (β = 0.096, p<0.05); Fig 5 shows the direct effect of authoritarian parenting style on preschoolers’ eating behavior was (β = 0.097, p<0.05);, ., which indicated that the four family parenting styles had significantly different effects on preschool children’s eating behavior. The differentiation of family dietary environment and parenting styles can lead to different eating behaviors and habits in children.

Currently, one-child families are the main model of Chinese families. Preschoolers’ eating habits are influenced by the domineering, authoritarian, and spoilt parenting methods that result from parents’ or other guardians’ overprotectiveness of young children. This phenomenon is highly consistent with the results of this study. In addition, this study found that only children scored higher on exogenous eating and emotional eating and lower on avoidance eating, which is consistent with the results of Wei, Xuan et al [34]. The preschool period is a critical stage of physical growth and development, and the development of good eating behaviors or the improvement of poor eating behaviors reflects the parents’ knowledge of preschool children’s dietary nutrition and family eating behaviors. Therefore, educating children’s guardians about healthy diets and teaching them skills in nutrition and eating behaviors can help improve preschool children’s eating behaviors, which is also consistent with the results of domestic and international studies [35].

### 4.3. Analysis of mediating effects of child survival quality, nutritional literacy

The mediating effect of eating behavior (CEBQ) under the influence of different family upbringing styles (EMBU) has a differential effect. In this study, we analyzed the status of the mediation effect of eating behavior (CEBQ) under the influence of family parenting style (EMBU) by comparing the results of Bootstrap mediation effect test of quality of survival (QLSCA) and nutritional literacy (NLS).

#### 4.3.1. Survival quality mediating effect analysis

Child survival quality is often defined as an individual’s comprehensive perception of illness [36], living conditions, and adaptive capacity. Current health-related survival quality in preschool children has been widely used to evaluate the effects of lifestyle population-level interventions in children and adolescents [37]. Family-related factors (e.g., whether they are the only child or not, family educational background, economic status, and parenting style) have a significant impact on the survival quality of preschool children. In this study, family parenting style influenced preschool children’s survival quality, and preschool children’s survival quality influenced their eating behavior habits, which shows that child survival quality plays a mediating role in the process of family parenting style influencing eating behavior [38]. The mediating effects of child survival quality on eating behavior under the influence of authoritative, coddling, authoritarian, and neglectful family parenting styles were [OR] 0.062, 95% CI 0.038–0.090; [OR] 0.056, 95% CI 0.031–0.088; [OR] 0.039, 95% CI 0.020–0.059; [OR] 0.025, 95% CI 0.010–0.044. It is evident that the quality of children’s survival status as well as their eating behaviors change under the effects of different family parenting styles [39]. It was found that the quality of survival for children was significantly better as they grew older and had higher levels of family education and socioeconomic status. In addition, families with relatively high nutritional and health quality parents are more likely to accept new healthy eating concepts and are willing to promote healthy lifestyles and good eating habits.

#### 4.3.2. Analysis of the mediating effect of nutritional literacy

Nutritional literacy is a specific type of health literacy, while health literacy is an advanced literacy in health-related settings. Carbone ET (2012) and Krause C (2018) concluded that nutritional literacy is a health literacy related to food choices and reflects an individual’s ability to access, process, and understand basic nutrition information and services and the ability to use this to make sound nutritional decisions [40, 41]. Nutritional literacy developed during the preschool years, a critical period for children’s growth and development, is directly related to food intake and eating behaviors [42]. Nutritional literacy in preschool children in this study includes three aspects of food and nutrition knowledge, concepts, and skills that are important hubs linking individuals, food, and environment and have predictive value for preschool children’s eating behavior [43]. The results of the Bootstrap mediation effect test of nutritional literacy showed that nutritional literacy (NLS) had a significant mediation effect between family parenting style (EMBU) and eating behavior (CEBQ). Among them, the mediating effects of eating behavior under the influence of authoritative, doting, authoritarian, and neglectful family parenting styles were [OR] 0.029, 95% CI 0.003–0.059; [OR] 0.026, 95% CI 0.002–0.054; [OR] 0.018, 95% CI 0.002–0.038; [OR] 0.012, 95% CI 0.001–0.028. It is evident that children’s nutritional literacy status as well as their eating behaviors change under the effects of different family parenting styles. Preschool children gradually enter the period of systematic learning, and through nutrition literacy education and other means, children’s unhealthy eating behaviors can be corrected, which is an important window period for children’s eating behavior change.

In addition, the results of the Bootstrap mediated effects test showed that nutritional literacy (QLSCA) and survival quality (NLS) had chain-mediated responses across different family parenting styles (EMBU) and eating behaviors (CEBQ). This means that families provide a specific environmental context for preschool children’s eating behaviors and energy intake, and the effects of different family domestic practices on children’s nutritional literacy and quality of survival are variable. Because preschool children’s lives and diets are primarily determined by their parents, the accumulation of family nutrition knowledge and the quality of their survival status will have a significant impact on the development of eating behavior [44]. Therefore, not only schools should strengthen nutrition and health education, but also families should pay attention to the cultivation of children’s eating habits, which is conducive to the development of good eating behaviors and habits in children and adolescents [45].

## 5. Conclusion

In the latest International Classification of Diseases, 11th edition (ICD-11), eating behavior problems in children refer to a series of problems such as feeding difficulties or eating difficulties exhibited by young children under adequate food supply and normal parental feeding, which then have great impact on the social, psychological, and physical functions of children. Therefore, it is important to explore the mechanism of developing eating behaviors and habits in preschool children from the perspective of different family parenting styles in order to improve children’s poor eating habits, cultivate good eating behaviors, and promote healthy growth in children and adolescents.

The study showed that family parenting style (EMBU) had a direct effect (-0.161 ≤ β ≤ 0.232, p < 0.05) and quality of survival (QLSCA) and nutritional literacy (NLS) had a significant mediating effect in this process, with a total indirect effect of [OR] 0.273, 95%:CI 0.181–0.368. Among them, the mediated effect value of quality of survival (QLSCA) on children’s eating behavior was 0.182 (p < 0.05) and the mediated effect value of nutritional literacy (NLS) on children’s eating behavior was 0.085 (p < 0.05). Therefore, the critical stage of preschool children should be seized to develop healthy eating behavior habits through proper family feeding practices.

This study also has some limitations, mainly: (1) the study is a cross-sectional study, using self-administered questionnaires, and the data collected at the same time period may have an impact on the mediating effect, making the parameters biased; (2) the factors affecting children’s eating behavior are more complex, such as family factors social factors and physiological factors, etc. This thesis mainly explores the influence of family factors on children’s eating behavior, because physiological factors This thesis mainly explores the influence of family factors on children’s eating behavior, and physical factors are not elaborated because they are not the focus of the topic. Therefore, the follow-up study needs to be further examined through longitudinal studies.

## Supporting information

S1 File (DOCX)
