# Supplementary material for: Influence of family parenting style on the formation of eating behaviors and habits in preschool children: The mediating role of quality of life and nutritional knowledge
Source: PLoS One. 2023 Jul 20;18(7):e0288878. doi: 10.1371/journal.pone.0288878 (PMC10358991; doi:10.1371/journal.pone.0288878)
Supplement: S1 File — (DOCX) [file pone.0288878.s001.docx]

**知情同意书**

尊敬的 先生/女士：

我们想邀请您参加一项《家庭教养方式对学龄前儿童饮食行为习惯养成的影响：生存质量和营养素养的中介作用》的研究，本研究已通过辽宁师范大学伦理委员会审核与批准。本知情同意书将向您介绍该研究的目的、步骤、获益、风险、不便以及您的权益等，请仔细阅读后慎重做出是否参加研究的决定。当研究人员向您说明和讨论知情同意书时，您可以随时提问并让他/她向您解释您不明白的地方。您可以与家人、朋友以及您的主治医师讨论之后再做决定。

知情同意声明：

我已被告知此项研究的目的、背景、过程、风险及获益等情况。我有足够的时间和机会进行提问，问题的答复我很满意。

我也被告知，当我有问题、想反映困难、顾虑、对研究的建议，或想进一步获得信息，或为研究提供帮助时，应当与谁联系。

我已经阅读这份知情同意书，并且同意参加本研究。

我知道我可以选择不参加此项研究，或在研究期间的任何时候无需任何理由退出本研究。

我已知道如果我的状况更差了，或者我出现严重的不良事件，或者我的研究医生觉得继续参加研究不符合我的最佳利益，他/她会决定让我退出研究。无需征得我的同意，资助方或者监管机构也可能在研究期间终止研究。如果发生该情况，医生将及时通知我，研究医生也会与我讨论我的其他选择。

我将得到这份知情同意书的副本，上面包含我和研究者的签名。

受试者姓名：________________________

受试者签名：________________________

她是自愿同意的。

研究者姓名：___ 王宁宁 程文广

研究者签名：_____
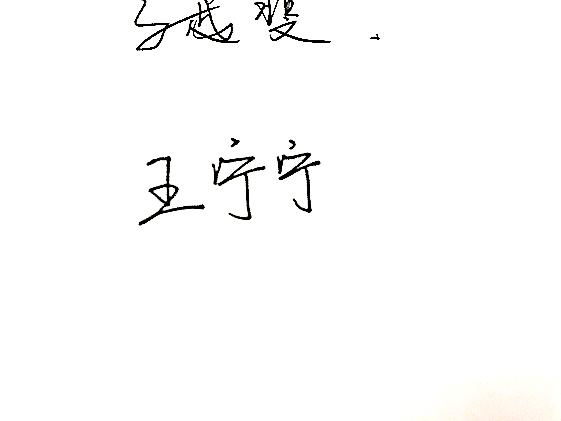
____
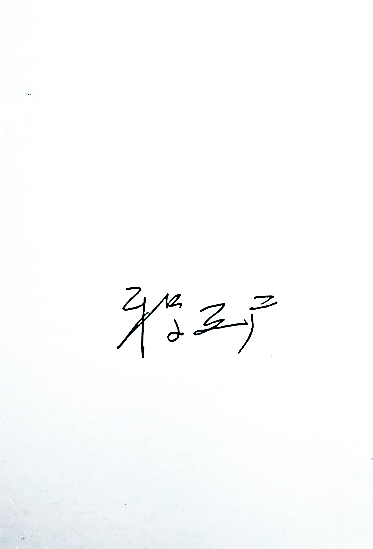
_____

日期：___2022____年___9_____月____10_____日

注：如果受试者不识字时尚需见证人签名，如果受试者无行为能力时则需代理人签
